# Job loss and health threatening events modulate risk-taking behaviours in the Covid-19 emergency

**DOI:** 10.1038/s41598-020-78992-x

**Published:** 2020-12-17

**Authors:** Caterina Galandra, Chiara Cerami, Gaia Chiara Santi, Alessandra Dodich, Stefano F. Cappa, Tomaso Vecchi, Chiara Crespi

**Affiliations:** 1 Istituti Clinici Scientifici Maugeri IRCCS, 27100 Pavia, Italy; 2 NEtS Center, Scuola Universitaria Di Studi Superiori IUSS-Pavia, Piazza della Vittoria, 15, 27100 Pavia, Italy; 3 Cognitive Computational Neuroscience Research Unit, IRCCS Mondino Foundation, 27100 Pavia, Italy; 4 Center for Neurocognitive Rehabilitation, CIMeC, University of Trento, Trento, Italy; 5 Dementia Research Center, IRCCS Mondino Foundation, 27100 Pavia, Italy; 6 Department of Brain and Behavioral Sciences, University of Pavia, 27100 Pavia, Italy

**Keywords:** Neuroscience, Human behaviour

## Abstract

Covid-19 pandemic is exerting a tragic impact all around the world. First-person experience of life-threatening and stressful events can modify individuals’ risk perception, and, consequently, risk-taking behaviours. Here we investigated risk-taking profiles in 130 Italian residents, and compared healthcare to non-healthcare workers, during the lockdown phase. We ad hoc developed the “Covid-19 Risk Task”, including the classic monetary Holt-Laury Paired Lottery Task (Monetary Condition, MC) and two new ecological conditions exploring Covid-19 related risk-taking aptitudes in relation to different health (Health Status Condition, HsC) and employment (Employment Status Condition, EsC) outcomes. Results showed that, in the whole sample, individuals were more risk-averse in MC than in HsC and EsC. Moreover, a payoff increase produced a shift toward more risk-averse behaviours in MC, but not in HsC and EsC, where we found an opposite trend suggesting a more risk-loving behaviour. Finally, we found that healthcare workers were significantly less risk-averse compared to non-healthcare workers in EsC, but not in MC and HsC. These findings provided evidence of the possible effects of Covid-19 outbreak on risk-taking aptitudes. The negative impact on human choices and, consequently, on the whole world economy of this catastrophic life event must not be underestimated.

## Introduction

Covid-19 pandemic has triggered in a few weeks a global health emergency and a social and economic crisis as never seen before. As the outbreak increased worldwide, forced measures of social distancing and isolation were progressively adopted by national governments. In Italy, the abrupt wave of infected cases recorded in Northern regions imposed extreme containment measures and social distancing from March to May 2020. In the meanwhile, there has been a complete reorganization of healthcare activities of public and private institutions in order to take charge of the high number of Covid-19 positive patients and to contain the spread of the epidemic, especially in Northern Italy.

Since the Italian government decree of March 9, 2020 (DCPM #iorestoacasa—I stay at home) imposed a lockdown all over the country, millions of people changed work habits, daily routines and lifestyles. Only a few industries, and small and large traders were kept open (i.e. manufacturers of essential goods or healthcare providers), while many workers have been signed for a temporary layoff, and in some cases dismissed. Freelance workers became suddenly unemployed. Millions of jobs are now at risk.

The huge number of deaths—i.e. 32.330 people at the time of writing (May 20, 2020)—that hit thousands of families, the dramatic economic consequences of the lockdown on job market (i.e. ~  + 15% of unemployed workers, job seekers or non-workers and ~ − 5% of Gross Domestic Product on the first quarter of 2020; https://www.istat.it/it/archivio/lavoro), and the decrease of people’s psychosocial well-being has exposed millions of Italian citizens to a large-scale catastrophic event, within a very short amount of time.

Data collected after large-scale catastrophic events as natural disasters (e.g., tsunami ^1^ or earthquake ^2^) or wars ^3^ proved that the first-person experience of extremely life-threatening and stressful events may change individuals’ perception of risk and consequently modulate their risk-taking aptitudes. Literature findings are, however, controversial about the direction of such a change. In some cases, the experience of extremely stressful catastrophe leads people to increase their risk perception, and thus they became more risk-averse. Risk-averse individuals typically prefer a sure outcome over a risky gamble, even in case of a higher expected value ^1–3^. Such an aptitude would keep the risk-averse subject safe from the experience of unpleasant feelings and negative emotions possibly emerging from an undesired outcome ^4^. For instance, Cassar and colleagues ^1^ showed that tsunami survivors were more risk-averse compared to subjects not directly involved in the catastrophic event. Despite the fact that only a few respondents declared that someone of their relatives was physically assaulted (3.7%) or had property stolen (5.8%) or burned (2.1%) during the civil protests, Jakiela and Ozier reported similar results in Kenyan young citizens after post-election crisis ^5^.

Conversely, other studies reported that the exposure to catastrophic events may produce a shift of the individual risk-taking profile toward a more risk-prone aptitude. In this case, risk-loving individuals prefer a more uncertain option over another with an equal but less risky expected outcome ^3,6^. Indeed, using a classical monetary task Eckel and colleagues ^6^ investigated individuals risk aptitude immediately after hurricane Katrina in New Orleans. They found that exposed subjects were on average more prone towards risk compared to a demographically matched group of Huston’s citizens not exposed to such a catastrophic event. A similar risk-proneness was observed in people gone through indiscriminate violence during civil-war between the two main ethnic groups of Burundi ^3^.

Taken together, all these findings confirm the idea that people who experienced first-person life-threatened catastrophic situations may undergo a significant change in their risk-taking aptitudes. They may thus become more risk-averse, as they experienced situations of extreme danger for their own lives and feel themselves as miracle survivors, or more risk-lovers, as they consider themselves immune to such danger.

No catastrophic event previously offered the opportunity to investigate changes in risk aptitudes directly during a large-scale long-lasting catastrophe. For this reason, Covid-19 outbreak is a unique opportunity for research. To the best of our knowledge, previous studies investigating risk-taking attitudes and behaviours in people who experienced catastrophic events typically used tasks with monetary stimuli and collected the data after the occurrence of such events, focusing on its long-term consequences. Moreover, the impact of catastrophic events on risk-taking aptitudes in relation to more ecological stimuli, including the possibility of falling ill or to suddenly change the employment status (e.g., layoffs, reductions in the working time), is still unexplored.

Taking into account the multifaceted consequences of the Covid-19 outbreak in Italy—including the sanitary emergency and its impact on psychosocial wellbeing, as well as the economic and job crisis—we developed a new task for risk-taking assessment (i.e. the Cov19 Risk Task, Cov19-RT), by adding two new ecological experimental conditions to the Holt-Laury Paired Lottery Task, a classical decision-making task based on a multiple price list (MPL) design including monetary stimuli ^7^. Thus, the Cov19-RT includes a Monetary Condition (Cov19-RT MC) and two conditions specifically designed to explore the individual risk-taking aptitudes in relation to different Covid-19 related health (Health Status Condition, Cov19-RT HsC) and employment (Employment Status Condition, Cov19-RT EsC) outcomes.

Our primary aim was thus to describe risk-taking profiles in Italian residents during the lockdown phase and record how the experience of Covid-19 pandemic may affect individuals’ risk-taking attitudes in the general population, by comparing the monetary condition to the new ecological ones. Then, we tested the presence of the so-called *incentive effect* (a general shift to the safe option due to a payoff increase, see ^7^) in all the conditions. In view of the very different employment conditions concerning healthcare compared to non-healthcare workers during the experimental observation time (i.e. a few weeks immediately after the lockdown when almost every job activities in Italy except for hospitals and healthcare facilities were closed), we investigated group differences on the basis of participants’ work status as well. This latter represents a variable of interest also in view of our previous report ^8^, which highlighted that healthcare workers perceived the Covid-19 emergency for health as more severe compared to non-healthcare workers.

In the current context, we expected that the ecological conditions related to health and employment outcomes—and specifically tailored on the Covid-19 pandemic—may provide a different, and possibly more realistic, description of risk-taking behaviours during a catastrophic event in comparison to the classical monetary condition. In particular, we hypothesized that Italians would be more averse toward risk in the monetary condition than in the health and employment ones. Finally, we expected that healthcare workers, who maintained their job occupation and salary during the Covid-19 pandemic, would be less risk averse in relation to the employment-related outcomes compared to non-healthcare workers.

## Results

### Demographic information and risk-taking profiles

**Figure 1:**
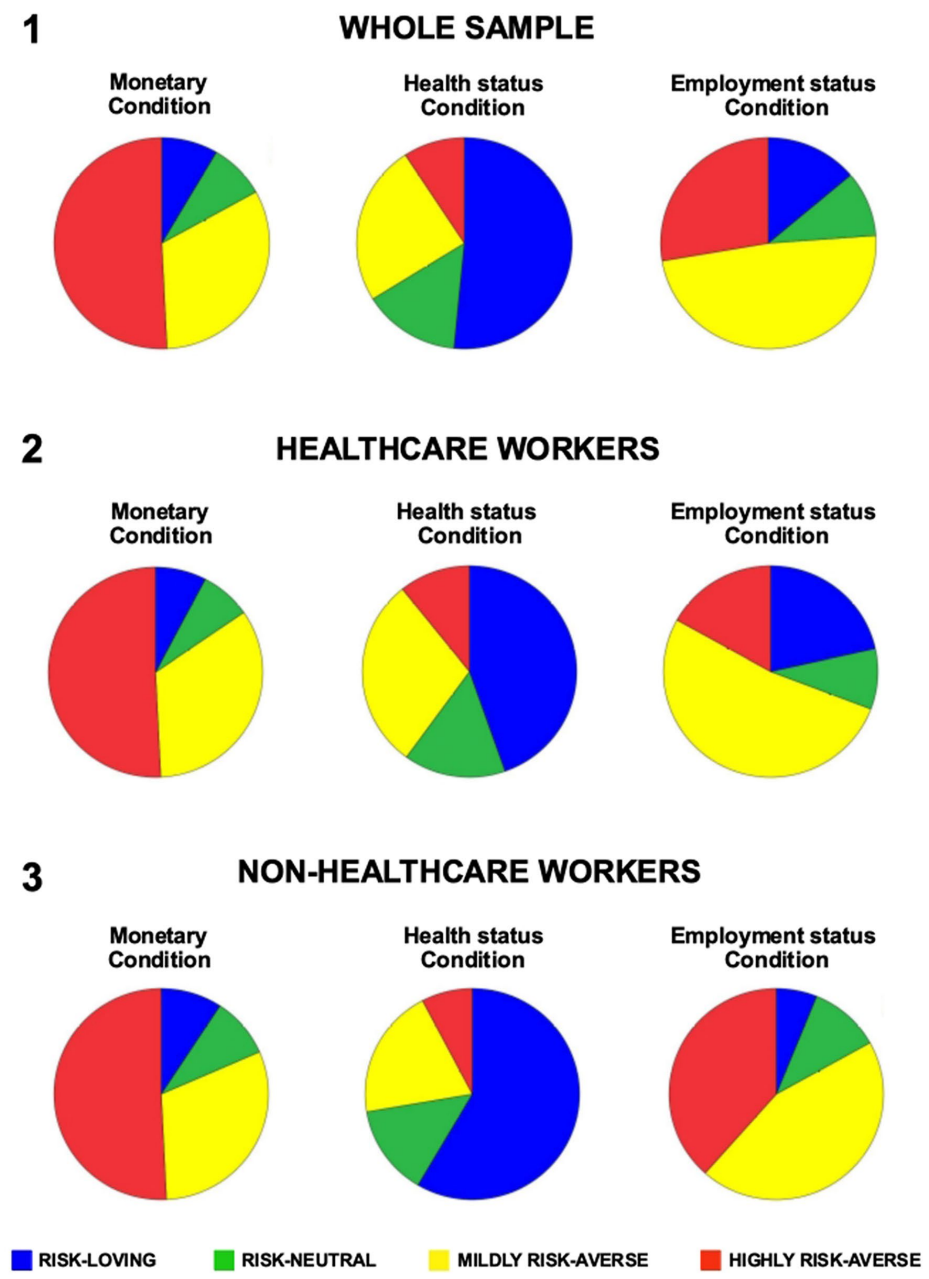
Classification of risk-taking profiles. The figure shows the proportion of different risk-taking profiles—i.e., risk-loving (blue sector), risk-neutral (green sector), mildly (yellow sector) and highly (red sector) risk-averse individuals—in the three Cov19-RT conditions considering the whole sample (n = 130), the healthcare workers group (n = 65) and non-healthcare workers group (n = 65).

Descriptive statistics of demographic information and risk-taking profiles are reported in Tables 1, 2 and Fig. 1.

**Table 1:** Demographic variables of the sample.

	Whole sample (n = 130)	Healthcare workers (n = 65)	Non-healthcare workers (n = 65)	p-value
Female/male %	68.5/31.5	83.1/16.9	53.8/46.2	< 0.001
Age in years (mean ± sd)	38.5 ± 9.3	38.4 ± 10.4	38.5 ± 8.2	0.985
Education in years (mean ± sd)	17.3 ± 1.4	17.5 ± 1.1	17.1 ± 1.7	0.114
Geographical area (Northern Italy/Southern-Central Italy) %	73.8/26.2	69.2/30.8	78.5/21.5	0.231

**Table 2:** Risk-taking profiles in the whole sample and in each group separately.

No. safe choices	CRRA range	Risk-taking aptitudes classification	Proportion of choices		
			Whole sample (n = 130)	Healthcare workers (n = 65)	Non-healthcare workers (n = 65)
**Monetary condition—mean values**					
0–3	− 0.95 < r > − 0.15	Risk-loving	8.5%	7.7%	9.2%
4	− 0.15 < r > 0.15	Risk-neutral	8.5%	7.7%	9.2%
5–6	0.15 < r > 0.68	Mildly risk-averse	32.2%	33.8%	30.8%
7–10	r > 0.68	Highly risk-averse	50.8%	50.8%	50.8%
**Health status condition—mean values**					
0–3	− 0.95 < r > − 0.15	Risk-loving	51.5%	44.6%	58.5%
4	− 0.15 < r > 0.15	Risk-neutral	14.6%	15.4%	13.8%
5–6	0.15 < r > 0.68	Mildly risk-averse	24.6%	29.2%	20%
7–10	r > 0.68	Highly risk-averse	9.3%	10.8%	7.7%
**Employment status condition—mean values**					
0–3	− 0.95 < r > − 0.15	Risk-loving	13.8%	21.5%	6.2%
4	− 0.15 < r > 0.15	Risk-neutral	10%	9.3%	10.7%
5–6	0.15 < r > 0.68	Mildly risk-averse	48.5%	52.3%	44.6%
7–10	r > 0.68	Highly risk-averse	27.7%	16.9%	38.5%

### Relationship between the monetary and the new ecological conditions

In the whole sample, we found a significant relationship between the two new ecological conditions (mHsC and mEsC: Spearman’s r = − 0.231, p < 0.008) but no significant relationship between the classical monetary condition and the new Covid-19 related conditions (mHsC and mMC: Spearman’s r = 0.47, p = 0.592; mEsC and mMC: Spearman’s r = 0.49, p = 0.578). Moreover, Freedman test on mMC, mHsC and mEsC highlighted significant differences in risk-taking behaviours between the three task conditions (**χ**^2^ = 76.177, p < 0.0001). Post hoc analysis (Wilcoxon signed-ranks test) revealed that subjects were significantly more risk-averse in mMC compared to both mHsC (z = − 7.919, p < 0.0001, effect-size: r = 0.895) and mEsC (z = − 3.002, p < 0.003, effect-size: r = 0.344), and in mEsC compared to mHsC (z = − 5.872, p < 0.0001, effect-size: r = − 0.663).

### Incentive effect

**Figure 2:**
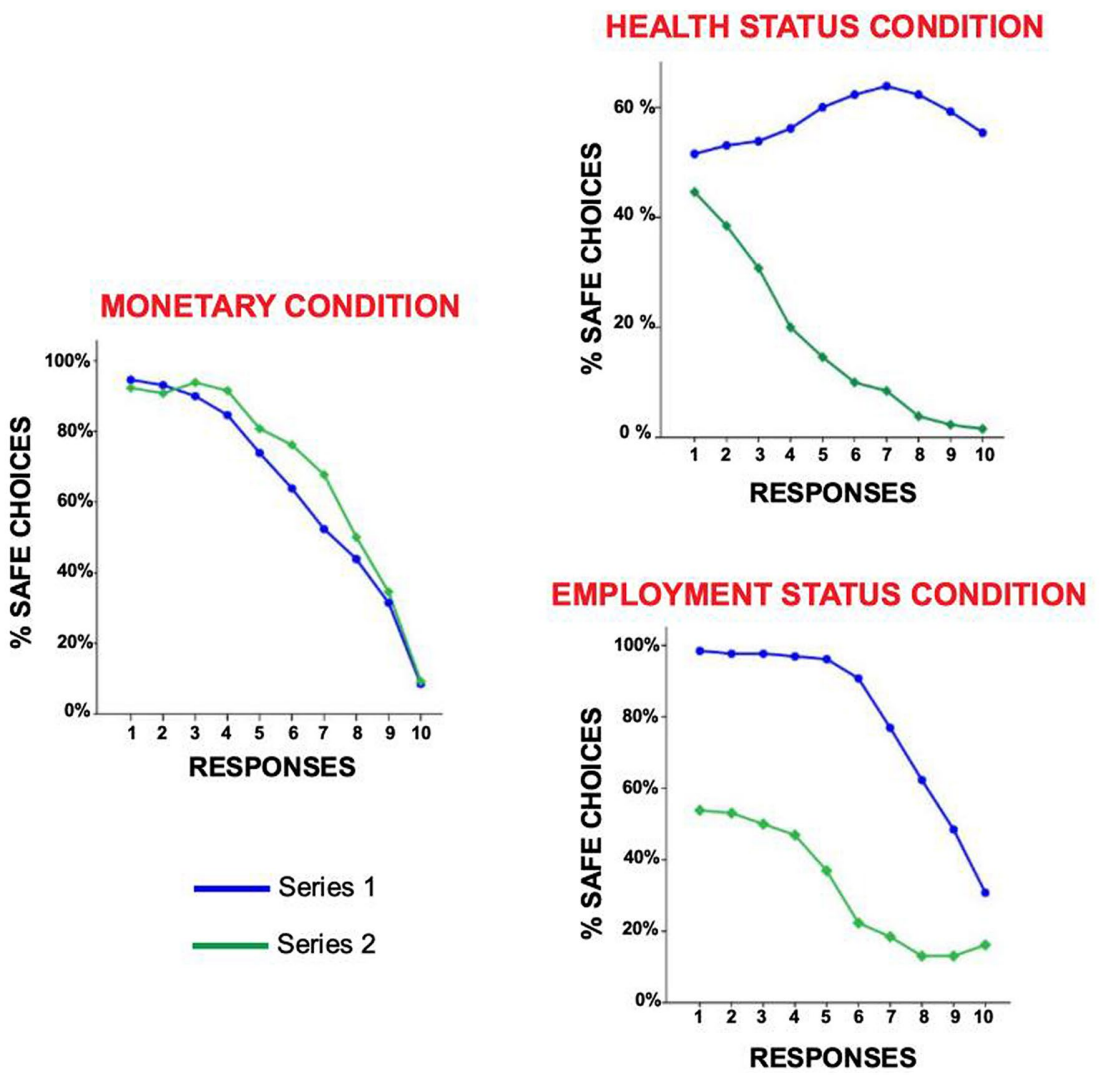
Incentive effect in the Cov19-RT conditions. The figure illustrates the incentive effect in the three different conditions in the whole group (n = 130), with the blue line representing the Series 1 (low payoff) and the green one depicting Series 2 (high payoff). In particular, we report, for each Series and condition, the average response in terms of percentages of safe choices (y-axis) for each of the 10 lottery rows (x-axis).

In order to test the presence of the incentive effect (i.e., a general shift to the safe option due to a payoff increase) we assessed a within-group comparison (Wilcoxon signed-rank test) on risk-taking profiles of Series 1 (lower payoffs) and Series 2 (higher payoffs). Wilcoxon signed-ranks test on Cov19-RT MC in the whole sample highlighted that a payoff increase from Series 1 to Series 2 produced a significant shift toward more risk-averse behaviours (z = − 3.343, p < 0.001, effect-size: r = − 0.532). In Cov19-RT HsC and EsC conditions we found the opposite trend, and the payoff increase produced a significant shift toward more risk-loving behaviours (HsC: z = − 7.780, p < 0.001, effect-size: r = 0.905; EsC: z = − 8.585, p < 0.001, effect-size: r = 0.921) (Table 3; Fig. 2).

**Table 3:** Incentive effect and risk aptitudes.

Incentive effect					
	z	p-value	r		
**Whole sample**					
mMC	− 3.343	0.001	− 0.532		
mHsC	− 7.880	< 0.001	0.905		
mEsC	− 8.585	< 0.001	0.921		
**Healthcare workers**					
mMC	− 1.040	0.298	− 0.233		
MHsC	− 5.477	< 0.001	0.907		
MEsC	− 6.042	< 0.001	0.910		
**Non-healthcare workers**					
mMC	− 3.699	< 0.001	− 0.830		
mHsC	− 5.666	< 0.001	0.898		
mEsC	− 6.139	< 0.001	0.932		

Differences in risk aptitudes					
	Healthcare workers (mean ± SD)	Non-healthcare workers (mean ± SD)	U	p-value	r
MC1	3.25 (± 0.985)	3.03 (± 1.060)	1880	0.242	− 0.110
MC2	3.25 (± 0.943)	3.45 (± 0.867)	2022.5	0.626	0.043
mMC	3.28 (± 0.910)	3.23 (± 0.965)	2080.5	0.870	− 0.015
HsC1	2.97 (± 1.224)	2.80 (± 1.227)	1943	0.401	− 0.080
HsC2	1.51 (± 1.017)	1.34 (± 0.796)	1993.5	0.434	− 0.056
mHsC	2.06 (± 1.088)	1.77 (± 1.027)	1795	0.108	− 0.150
EsC1	3.57 (± 0.756)	3.89 (± 0.437)	1562	< 0.001	0.261
EsC2	1.82 (± 1.102)	2.20 (± 1.148)	1704	0.040	0.193
mEsC	2.642 (± 1.007)	3.154 (± 0.854)	1517.5	0.003	0.282

MC1 = monetary condition series 1, MC2 = monetary condition series 2, mMC = mean monetary condition, HsC1 = health condition series 1, HsC2 = health condition series 2, mHsC = mean health condition, EsC1 = employment condition series 1, EsC2 = employment condition series 2, mEsC = mean employment condition, z = Wilcoxon test, r = rank-biserial correlation, U = Mann Whitney, SD = standard deviation.

When we considered the healthcare and non-healthcare workers separately, we found that both groups displayed the incentive effect in HsC (healthcare workers: z = − 5.477, p < 0.001, effect-size: r = 0.907; non-healthcare workers: z = − 5.666, p < 0.001, effect-size: r = 0.898) and EsC (healthcare workers: z = − 6.042, p < 0.001, effect-size: r = 0.910; non-healthcare workers: z = − 6.139, p < 0.001, effect-size: r = 0.932), but only non-healthcare workers showed a significant payoff effect in MC (z = − 3.699; p < 0.001, effect-size: r = − 0.830) (Table 3).

### Risk-taking aptitudes in healthcare versus non-healthcare workers

Between-group analysis (Mann–Whitney test) comparing healthcare and non-healthcare workers on risk-taking aptitude profiles highlighted significant differences in EsC measures (EsC1: U = 1562, p < 0.0001, effect-size: r = 0.261; EsC2: U = 1704, p < 0.040, effect-size: r = 0.193; mEsC: U = 1517.5, p < 0.003, effect-size: r = 0.282), showing that healthcare workers are significantly less risk-averse compared to non-healthcare workers (Table 3). We did not found significant group differences in MC (MC1: U = 1880, p < 0.242, effect-size: r = − 0.110; MC2: U = 2022.5, p < 0.626, effect-size: r = 0.043; mMC: U = 2080.5, p < 0.870, effect-size: r = − 0.015) and HsC (HsC1: U = 1943, p < 0.401, effect-size: r = − 0.080; HsC2: U = 1993.5, p < 0.434, effect-size: r = − 0.056; mHsC: U = 1795, p < 0.108, effect-size: r = − 0.150) measures. See Table 3.

## Discussion

Italy was the first Western country to be severely affected by the SARS-CoV2 virus. The way Italian people are reacting to this life-threatening event, as well as to the severe restrictions imposed on work activities, likely reflects the disruptive changes that the Covid-19 outbreak, first, and the lockdown measures, later, have exerted on health and economic outcomes in Italy.

In this study, we explored real-life behaviours in the days of the Covid-19 outbreak trying to expand current literature about individual risk-taking aptitudes and following behaviours in relation to the experience of catastrophic events that may put life in danger and jobs at risk. We also aimed to provide useful insights to orient healthcare and economy strategies in the very next future and in case of novel pandemic.

As the Covid-19 lockdown measures were put in place, the first impulse was to make a historical parallel. However, as the weeks went by, the singularity of the shock we are going through became clear. The numbers of deaths seen over such a short time, and the current implosion of the world economy concentrated within a few months, make this pandemic an exceptional event. Literature suggests that the first-person experience of extremely life-threatening and stressful events usually can lead people to increase their risk perception, pushing individuals to became more risk-averse ^1,5^. However, there is also opposite evidence reporting that catastrophe survivors display more risk-loving aptitude ^3,6^. These opposite profiles in risk-taking aptitudes after the experience of life-threatening events have been inferred from experimental settings using risk tasks with monetary lotteries. Moreover, these tasks have been administered to survivors only after the catastrophic events ^1,3,5,6^.

In the light of these considerations, it is likely that both the late timing of data collection and the use of paradigms including exclusively monetary stimuli—which might be not suitable enough to capture real-life changes—somewhat biased previous inferences on risk aptitude profiles of survivors from catastrophic events. The use of more ecological stimuli, as those developed for this study, together with a well-timed data collection during the emergency period, can possibly overcome such limitations.

Our data showed that, overall, individuals displayed more risk-averse behaviours in the monetary condition than in the novel ecological ones. Notably, while individual patterns observed in the two ecological conditions were significantly correlated, we did not find significant association with risk-taking profiles emerging from the classic monetary condition. This evidence suggests that the individual variability in risk-taking profiles emerging from the monetary condition is totally independent from that resulting from the Covid-19-related conditions. These two latter, exploring individuals’ risk-taking aptitudes in two very different domains, namely health and employment status, are tightly and negatively linked to each other. Notably, the negative direction of the relationship between the two ecological conditions might be explained in terms of a preferential allocation of attentional resources to the most relevant domain for the individual during the Covid-19 outbreak. To this purpose, we could speculate that people who are more worried about possible long-term economic consequences, as a result of changes possibly occurring in their employment condition, would display a more risk-averse aptitude in the EsC rather than in the HsC, as they are possibly more willing to undertake the risk to fall ill rather than the risk to experiment aversive economic consequences due to the detrimental impact of Covid-19 outbreak on job market and productive activities. On the contrary, this evidence might suggest that who is more concerned about possible negative health outcomes related to the Covid-19 outbreak, would then show a more risk-averse aptitude in the HsC rather than in the EsC, as the worry about health overcomes that of losing a regular income, or also because Covid-19 emergency did not affect their job occupation.

Moreover, in line with the literature ^7^, we observed the presence of a significant *incentive effect* in MC, with a shift toward more risk-averse behaviours as the payoff increased. As expected, Italians inhabitants, who are actually experiencing such an unpredictable life situation, possibly afflicting each kind of people’s certainties, preferred a sure monetary outcome over a risky gamble. A risk-averse aptitude may, indeed, work as a self-protection strategy, keeping people safe from the experience of unpleasant feelings and negative emotions emerging from an undesired but possible negative monetary outcome.

At the opposite, we observed a *reversed incentive effect* by comparing Series 1 and Series 2 payoffs in HsC and EsC. Indeed, in both these conditions, we found a shift toward more risk-loving behaviours as the payoff increased. This evidence may be explained by the specific nature of the ecological lotteries, which allow people to precisely anchor each stimulus to possible health and employment consequences related to the Covid-19 emergency, and thus resulting particularly salient for the self, compared to more abstract monetary stimuli. Indeed, since these stimuli encase real outcomes defining individuals’ health, socio-economical and, ultimately, personal status, risk-loving behaviours might be due to the attempt to reach the most favourable condition and/or to avoid the most potentially threatening ones. Moreover, by including prevalently young adults (mean age = 38 y.o.; median = 37 y.o.), it is possible that such a *reversed incentive effect* may reflect the fact that they consider themselves in an advantageous condition, having a great number of opportunities for their personal life and, thus, willing to risk in order to obtain the best outcome possible. Moreover, younger people have been proved to have more risk-prone aptitudes than older people ^9^.

Overall, our results on the incentive effect in MC condition confirmed Holt and Laury ^7^ findings, highlighting that scaling up the payoff in *hypothetical* monetary lotteries (i.e. without a real winning) makes little differences in risk-taking aptitudes. Conversely, using ecological stimuli specifically related to the current emergency situation has the same effect as *real* monetary lotteries (i.e. with a real winning ^7^) provoking a greater shift in risk-taking aptitudes, as proved by a larger effect size in HsC and EsC compared to MC (see Fig. 2). Thus, it seems that Covid-19 related ecological stimuli, because of their intrinsic features triggering real-life experiences, are able to elicit more realistic risk-related behaviours.

Finally, we found that healthcare workers were significantly less risk-averse compared to non-healthcare workers in the EsC conditions, but not in the MC and HsC conditions. This finding may reflect the overall stability of healthcare workers’ employment status, differently from other workers whose occupation was actually at risk or unpredictable, due to the lockdown measures. Indeed, the unemployment rate—which was at an all-time low even in countries like the United States—is exponentially growing now. Western economies have been hit by an unprecedented shock wave. Economic cycles usually start from the most unstable sectors (such as real estate or construction) or from the most exposed to global competitors (e.g. automotive industry). This time, however, the lockdown also affected retail services, education, the entertainment industry, as well as restaurants, tourism and travel sectors. All these activities employ the majority of a country's population. For this reason, the catastrophic effect has been immediate, and millions of jobs will be permanently lost. Such a negative view influence individuals’ risk perception, likely prompting more risk-averse aptitudes in approaching disruptive consequences affecting the working world.

The first major limitation of the present study is represented by the cross-sectional nature of the report, which prevents to draw causal conclusions on longitudinal changes of individual risk-taking aptitudes. Moreover, the small-to-moderate sample size and the unbalanced male/female ratio in healthcare versus non-healthcare workers—due to the convenience sampling of PsyCOVID study ^8^—may hinder the generalization of present results to the general population. Another limitation of the study is the lack of controlling on the geographical origin of participants, preventing us to infer robust information about how risk attitude has been affected by the spread of epidemic. Only future replication studies using both monetary and ecological conditions on larger samples and with detailed information about geographical origin of participants can confirm the reliability of the present results.

In conclusion, we provided evidence that experiencing the psychosocial consequences of Covid-19 outbreak can modulate individual risk-taking profiles in real-life situations. The tragic negative effects of this catastrophic life event, threatening health and occupation must not be underestimated.

## Methods

### Sample size

We carried out an *‘*a priori*’* power analysis on the risk aptitude difference between the three conditions of the Covid-19 Risk Task (Cov-19 RT). We used G*Power (https://g-power.apponic.com/) and found that a sample size of 128 subjects provides a statistical power of 0.95 in identifying a significant difference, performing a Wilcoxon signed-ranks test (matched pairs) with effect size dz = 0.3 and statistical significance of 0.05.

### Participants

Immediately after the lockdown phase disposed by Italian government on March 9, 2020 (DCPM #iorestoacasa—I stay at home), we launched the PsyCOVID study (https://wprn.org/item/428452) aiming at evaluating changes in lifestyles, habits, routines and psychosocial dimensions in the Italian population during the social distancing period (see baseline findings at ^8^).

The original PsyCOVID Study sample included 165 healthcare workers out of 1163 participants ^8^. In order to consider only people of working age and compare healthcare workers with non-healthcare workers we applied the following exclusion criteria: (1) age below 25 years old and over 65 years old, (2) working status, namely we excluded who formerly recorded themselves as unemployed, student, housewife or retired. We then obtained a healthcare workers sub-sample including 140 participants. Thus, we randomly selected 140 participants among non-healthcare workers of the PsyCOVID Study dataset, who were matched for age, education, gender and geographic area (Northern or Central-Southern Italy). We finally sent the invitation to the Covid-19 Risk Task to these 280 participants.

We implemented the experimental task Cov19-RT in an on-line survey with Google Forms, which we distributed by direct link, via e-mail or Whatsapp to each participant. Participants provided their written informed consent to the experimental procedure, which was approved by the IUSS-University of Pavia Ethics Committee.

Between April 3 and 23, 2020, 65 healthcare workers (females: 83.1%; mean age: 38.43 ± 10.37 yo; age range: 25–64 yo; mean years of education: 17.51 ± 1.11 yy; education range: 13–18 yy) and 65 non-healthcare workers (females: 53.8%; mean age: 38.46 ± 8.18 y.o.; age range: 25–64 y.o.; mean years of education: 17.11 ± 1.7 yy; education range: 13–18 yy) fully completed the task. The response rate was 46%. We calculated the response rate as the ratio of the number of fully completed surveys to the total number of potential participants (i.e., 280) who were invited to take part in the study. Healthcare and non-healthcare worker groups resulted matched for all the considered variables (age, education, geographic area), except for sex. Table 1 provides details about the participants’ socio-demographic characteristics.

### The Covid-19 risk task

In order to assess risk-taking aptitudes during Covid-19 pandemic, we created a modified version of the Holt-Laury Paired Lottery Task ^7^ with hypothetical monetary payoff, a task wildly used to assess risk-related behaviours after catastrophic events, such as natural disasters ^10^. In the original task, composed by 10 paired monetary lotteries presented on different decision rows, participants have to make a choice between Lottery A and Lottery B (Table 4). In any decision row, Lottery A always shows the “safe” choice while Lottery B represents the “risky” choice, as Lottery A has less variability in the payoffs than Lottery B. The 10 decision rows differed in terms of the probability of winning the higher prize in each lottery. In the first decision row, the probability of winning the higher prize is 10%, while for the subsequent 9 decision rows, the probability to obtain the better outcome progressively increases by 10% so that by decision row 9 there is a 90% chance of winning the higher prize, and decision row 10 is a choice between two certain winnings.

**Table 4:** Series 1 items of Lottery A and Lottery B for the three Covid-19 risk task conditions.

Covid-19 risk task monetary condition			
Decision Raw	Lottery A	Lottery B	CRRA range
1	10% 200 €–90% 160 €	10% 385 €–90% 10 €	r < − 0.95
2	20% 200 €–80% 160 €	20% 385 €–80% 10 €	− 0.95 < r > − 0.49
3	30% 200 €–70% 160 €	30% 385 €–70% 10 €	− 0.49 < r > − 0.15
4	40% 200 €–60% 160 €	40% 385 €–60% 10 €	− 0.15 < r > 0.15
5	50% 200 €–50% 160 €	50% 385 €–50% 10 €	0.15 < r > 0.41
6	60% 200 €–40% 160 €	60% 385 €–40% 10 €	0.41 < r > 0.68
7	70% 200 €–30% 160 €	70% 385 €–30% 10 €	0.68 < r > 0.97
8	80% 200 €–20% 160 €	80% 385 €–20% 10 €	0.97 < r > 1.37
9	90% 200 €–10% 160 €	90% 385 €–10% 10 €	r > 1.37
10	100% 200 €–0% 160 €	100% 385 €–0% 10 €	r > 1.37

Covid-19 risk task health status condition			
Decision raw	Lottery A	Lottery B	CRRA
1	10% Symptomatic Covid-19 infection without hospitalization—90% Type II Diabetes Mellitus	10% Shoulder Fracture—90% Symptomatic Covid-19 infection with hospitalization	r < − 0.95
2	20% Symptomatic Covid-19 infection without hospitalization—80% Type II Diabetes Mellitus	20% Shoulder Fracture—80% Symptomatic Covid-19 infection with hospitalization	− 0.95 < r > − 0.49
3	30% Symptomatic Covid-19 infection without hospitalization—70% Type II Diabetes Mellitus	30% Shoulder Fracture—70% Symptomatic Covid-19 infection with hospitalization	− 0.49 < r > − 0.15
4	40% Symptomatic Covid-19 infection without hospitalization—60% Type II Diabetes Mellitus	40% Shoulder Fracture—60% Symptomatic Covid-19 infection with hospitalization	− 0.15 < r > 0.15
5	50% Symptomatic Covid-19 infection without hospitalization—50% Type II Diabetes Mellitus	50% Shoulder Fracture—50% Symptomatic Covid-19 infection with hospitalization	0.15 < r > 0.41
6	60% Symptomatic Covid-19 infection without hospitalization—40% Type II Diabetes Mellitus	60% Shoulder Fracture—40% Symptomatic Covid-19 infection with hospitalization	0.41 < r > 0.68
7	70% Symptomatic Covid-19 infection without hospitalization—30% Type II Diabetes Mellitus	70% Shoulder Fracture—50% Symptomatic Covid-19 infection with hospitalization	0.68 < r > 0.97
8	80% Symptomatic Covid-19 infection without hospitalization—20% Type II Diabetes Mellitus	80% Shoulder Fracture—20% Symptomatic Covid-19 infection with hospitalization	0.97 < r > 1.37
9	90% Symptomatic Covid-19 infection without hospitalization—10% Type II Diabetes Mellitus	90% Shoulder Fracture—10% Symptomatic Covid-19 infection with hospitalization	r > 1.37
10	100% Symptomatic Covid-19 infection without hospitalization—0% Type II Diabetes Mellitus	100% Shoulder Fracture—0% Symptomatic Covid-19 infection with hospitalization	r > 1.37

A “risk-neutral” individual usually selects Lottery A for the first four choices, either A or B for choice 5 (i.e. 50–50%) and then switches over Lottery B for the last four choices. Considering the utility function

$${\text{u}}\left( {\text{x}} \right) = {\text{x}}^{{{\text{r}} - {1}}}$$

where *x* represents the prize and *r* represents the constant relative risk aversion coefficient (CRRA) ^7,9^, risk-neutral conditions are defined by *r* = 0, while risk-loving and risk-averse conditions by, respectively, *r* > 0 and *r* < 0. In the present work, we characterized the individual risk-taking profile on the basis of Albert’s *r* cut-offs ^9^. We considered four types of risk-taking profiles. Identifying participants’ as “risk-loving” (*r* < − 0.15, 0–3 safe choices), “risk-neutral” (− 0.15 < *r* > 0.15, 4 safe choices), “mildly risk-averse” (0.15 < *r* > 0.68, 5–6 safe choices) and “highly risk-averse” (*r* > 0.68, 7 or more safe choices).

In addition to the classic monetary condition of Holt-Laury Paired Lottery Task (i.e. Cov19-RT MC), the Cov19-RT included two novel ecological Covid-19 related conditions, with lotteries entailing health- and employment-related outcomes (i.e. Cov19-RT HsC and EsC). Each condition (i.e. MC, HsC and EsC) included two Series of 10 paired lotteries with different payoff amounts (i.e. Series 1 lower payoffs and Series 2 higher payoffs) with the aim of investigating if different payoff amounts could elicit different risk behaviours. In order to avoid a confounding effect by comparing real monetary vs. hypothetical health and employment payoffs, we included only hypothetical payoffs in the three conditions (i.e. monetary, health and employment) of the task.

Cov19-RT HsC and EsC were developed with the same design as MC, including paired lotteries of pathological conditions and employment status reflecting outcomes possibly occurring during the lockdown imposed by the Covid-19 emergency as choice alternatives.

Preliminary to the administration of the task we performed a content validity procedure for the novel ad hoc developed materials to be included in the two novel ecological conditions ^11^. Specifically, we created two lists of 24 items (i.e. pathological conditions and employment status) (see Supplementary Tables for item lists) and we asked 38 healthy subjects, balanced for gender and age groups (i.e. 20–70 yo), to assess content validity of the novel experimental conditions. First, we asked subjects to assess face validity (e.g. understanding of sentences and presentation of the text). Then, to rate each item in terms of illness severity or employment hardship on a 5-point Likert scale (e.g., 0 = Not serious at all; 1 = Not very serious; 2 = Serious enough; 3 = Serious; 4 = Extremely serious). We then excluded unclear items and ordered the remaining items, based on overall severity perception. Finally, we designed Lottery A and Lottery B with the same CRRA coefficient of the monetary condition (i.e., health and employment conditions perceived as less severe were associated with the higher monetary values, while health and employment conditions perceived as more severe were associated with the lower monetary values), as reported in the previous paragraph.

Thus, while Cov19-RT MC includes monetary outcomes (Series 1 Lottery A: 200€-160€, Lottery B: 385€–10€; Series 2 Lottery A: 4.000€–3.200€, Lottery B: 7.700€–200€), HsC entails health outcomes (Series 1 Lottery A: Symptomatic SARS-CoV2 infection without hospitalization—Type II Diabetes Mellitus, Lottery B: Shoulder Fracture—Symptomatic SARS-CoV2 infection with hospitalization; Series 2 Lottery A: Psoriasis—Asymptomatic SARS-CoV2 infection, Lottery B: Cold—Symptomatic SARS-CoV2 infection without hospitalization) and EsC employment outcomes (Series 1 Lottery A: Paid leave (salary reduced of 2/3)—Work from home with 50% salary reduction, Lottery B: 30 days of paid leave—Unpaid furlough; Series 2 Lottery A: 15 days of paid leave—Work with reduced work hours (part-time), Lottery B: Work from home with full salary—Paid leave (salary reduced of 2/3)).

Preliminary to the task administration, we provided detailed instructions and examples for each condition (see Supplementary Materials).

See Tables 4 for details on Cov19-RT stimuli.

### Statistical analyses

We carried out statistical analyses using SPSS (https://www.spss.it/) and JASP (https://jasp-stats.org/), using non-parametric tests. We set statistical significance at p < 0.05 for all statistical tests performed.

#### Demographic and risk-taking aptitude variables

We performed descriptive statistics on (i) Demographic variables, reporting mean and standard deviation for pseudo-continuous measures and frequency and percentage for categorical descriptors; and (ii) Risk-taking aptitudes, reporting frequency and percentage of different risk profiles (risk-loving, risk-neutral, mildly risk-averse, highly risk-averse).

For this purpose, we calculated a risk-taking measure (i.e. MC, HsC and EsC) for each Series (1 and 2) within each condition, using the number of safe choices (Lottery A), i.e. MC-Series 1 (MC1), MC-Series 2 (MC2), HsC-Series 1 (HsC1), HsC-Series 2 (HsC2), EsC-Series 1 (EsC1) and EsC-Series 2 (EsC2).

In addition, we computed an overall risk-taking aptitude measure for each condition (average of safe choices among the two Series), i.e., Mean MC (mMC), Mean HsC (mHsC), Mean EsC (mEsC).

The description of demographic information and risk-taking profiles are reported in Tables 1 and 2.

#### Risk-taking behaviours

In order to assess the overall functioning of the two new ecological Covid-19 related conditions (HsC and EsC) compared to the classical monetary condition (MC), we calculated the Spearman’s Rank correlation coefficient among mMC, mHsC and mEsC in the whole sample.

Then, for each condition, we assessed a within-group comparison (Wilcoxon signed-rank test) on risk-taking profiles of Series 1 (lower payoffs) and Series 2 (higher payoffs) to test the presence of the *incentive effect* (a general shift to the safe option due to a payoff increase, see ^7^). We performed this analysis in the whole group, as well as in healthcare and non-healthcare worker groups.

Moreover, we investigated within-group differences (Freedman Test and Wilcoxon post hoc analysis) in risk-taking profiles among the three conditions of the task (mMC, mHsC, mEsC).

Finally, we explored between-group differences in risk-taking profiles in healthcare workers vs. non-healthcare workers (Mann–Whitney U Test) in each Series within each condition (MC1, MC2, HsC1, HsC2, EsC1, EsC2), as well as in global measures (mMC, mHsC, mEsC). For each test providing significant results we calculated effect size (Glass’s rank biserial correlation test for non parametric statistics).

### Ethical approval

The present work is part of the PsyCOVID Study (https://wprn.org/item/428452), which was approved by the IUSS-University of Pavia Ethics Committee. All participants provided their written informed consent to take part in the study. All procedures were in accordance with the ethical standards of local Committee of Human Experimentation, and with the Helsinki Declaration of 1975 as revised in 2000.

## Supplementary information

Supplementary Information
