# Supplementary material for: Job loss and health threatening events modulate risk-taking behaviours in the Covid-19 emergency
Source: Sci Rep. 2020 Dec 17;10:22236. doi: 10.1038/s41598-020-78992-x (PMC7746716; doi:10.1038/s41598-020-78992-x)
Supplement: Supplementary file 1 — Supplementary Information [file 41598_2020_78992_MOESM1_ESM.docx]

**SUPPLEMENTARY INFORMATION**

**Job loss and health threatening events modulated**

**risk-taking behaviours in the Covid-19 emergency**

*Authors*

Caterina Galandra, Chiara Cerami, Gaia Chiara Santi, Alessandra Dodich, Stefano F. Cappa, Tomaso Vecchi, Chiara Crespi

**Supplementary Table S1** – List of items used to create the Health status Condition (HsC) of the Covid-19 Risk Task and relative rating scores.

| **Severity perception** | **HsC item options**  **(pathological condition)** | **Rating**  **(average data)** |
| --- | --- | --- |
| 1  (less severe condition) | Cold | 1.21 |
| 2 | Sore throat | 1.50 |
| 3 | Cough | 1.71 |
| 4 | Diarrhea | 1.76 |
| 5 | Hemorrhoids | 1.76 |
| 6 | Headache | 1.92 |
| 7 | Gastroesophageal reflux | 2.08 |
| 8 | Cystitis | 2.13 |
| 9 | Allergy | 2.21 |
| 10 | Psoriasis | 2.24 |
| 11 | Shoulder fracture | 2.42 |
| 12 | Asymptomatic Covid-19 infection | 2.63 |
| 13 | Arthrosis | 2.82 |
| 14 | Bronchial asthma | 2.84 |
| 15 | Osteoporosis | 2.95 |
| 16 | Thighbone fracture | 3 |
| 17 | Symptomatic Covid-19 infection without hospitalization | 3 |
| 18 | Diabetes mellitus | 3.53 |
| 19 | Symptomatic Covid-19 infection with hospitalization | 4.05 |
| 20 | AIDS | 4.37 |
| 21 | Alzheimer’s disease | 4.37 |
| 22 | Lung cancer | 4.45 |
| 23 | Heart attack | 4.47 |
| 24  (most severe condition) | Stroke | 4.53 |

**Supplementary Table S2** – List of items used to create the Health status Condition (HsC) of the Covid-19 Risk Task and relative rating scores.

| **Severity perception** | **EsC item options (employment status)** | **Rating**  **(average data)** |
| --- | --- | --- |
| 1  (less severe condition) | Work from home with full salary | 1.31 |
| 2 | Paid leave (full salary) | 1.71 |
| 3 | Work with paid overtime | 1.94 |
| 4 | 5 days of paid leave | 2 |
| 5 | Work full salary + bonus from government support (DPCM-CuraItalia) | 2 |
| 6 | Work with standard salary | 2.08 |
| 7 | 15 days of paid leave | 2.16 |
| 8 | Freelance job (a third of income + bonus from government support, DPCM-CuraItalia) | 2.16 |
| 9 | 10 days of paid leave | 2.26 |
| 10 | 15 sick-leave days | 2.29 |
| 11 | 10 sick-leave days | 2.34 |
| 12 | Work without meal voucher | 2.37 |
| 13 | Work with reduced work hours(part-time) | 2.58 |
| 14 | 30 sick-leave days | 2.63 |
| 15 | 30 days of paid leave | 2.66 |
| 16 | Paid leave (salary reduced 50%) | 2.66 |
| 17 | Paid leave (salary reduced of 2/3)) | 2.92 |
| 18 | ”Cassa Integrazione” (paid furlough) | 3.08 |
| 19 | Work from home with 50% salary reduction | 3.18 |
| 20 | Work withunpaid overtime | 3.29 |
| 21 | Unpaid furlough | 3.74 |
| 22 | Freelance with reduced work hours(2/3 reduction) with no government support | 3.92 |
| 23 | Unpaid work from home | 4.26 |
| 24  (most severe condition) | Layoff | 4.39 |

**Supplementary Methods**

***Monetary Condition Instruction***

In the next section you will find two series of stimuli, each one including 10 hypothetical lotteries. Gambling these lotteries you may virtually win money. Each lottery includes two options (A and B) differing for both the amount (in euro) of the possible winning and for the probability (in percentage) to obtain that outcome.

EXAMPLE:

OPTION A: 10% to earn 20 € - 90% to earn 16 €.

OPTION B: 10% to earn 35 € - 90% to earn 1 €.

In this case, if you choose OPTION A you will have 10% of possibility to earn 20 € and the 90% of possibility to earn 16 €. Otherwise, if you choose OPTION B, you will have 10% to earn 35 € and 90% to earn 1 €.

Thus, for each of the 10 lotteries you have to choose one of the two options, A or B. You can answer in the order that you prefer. You can revise and change your answer before to confirm and step to the next series of lotteries. Remember, there is not a right or a wrong answer.

***Health status Condition Instruction***

In the next section you will find two series of stimuli, each one including 10 hypothetical lotteries. Gambling these lotteries you may virtually get a disease. Each lottery includes two options (A and B) differing for both the type of pathological condition and for the probability (in percentage) to obtain that outcome.

EXAMPLE

OPTION A: 10% of getting asthma – 90% of getting osteoporosis

OPTION B: 10% of getting cough – 90% of getting a shoulder fracture

In this case, if you choose OPTION A you will have 10% of possibility of getting asthma and the 90% of possibility of getting osteoporosis. Otherwise, if you choose OPTION B, you will have 10% of getting cough and 90% of getting a shoulder fracture.

Thus, for each of the 10 lotteries you have to choose one of the two options, A or B. You can answer in the order that you prefer. You can revise and change your answer before to confirm and step to the next series of lotteries. Remember, there is not a right or a wrong answer.

***Employment status Condition Instruction***

In the next section you will find two series of stimuli, each one including 10 hypothetical lotteries. Gambling these lotteries you may virtually run into different working conditions related the current pandemic. Each lottery includes two options (A and B) differing for both the working condition and for the probability (in percentage) to obtain that outcome.

EXAMPLE

OPTION A: 10% to be 5 days off – 90% of working without having meal vouchers

OPTION B: 10% to be paid leaved (full salary) – 90% to be fired

In this case, if you choose OPTION A you will have 10% of possibility of being 5 days off and the 90% of possibility of working without having meal vouchers. Otherwise, if you choose OPTION B, you will have 10% to be paid leaved (full salary) and 90% to be fired.

Thus, for each of the 10 lotteries you have to choose one of the two options, A or B. You can answer in the order that you prefer. You can revise and change your answer before to confirm and step to the next series of lotteries. Remember, there is not a right or a wrong answer.
